# Intravenous calcium Gluconate alleviates Lead-induced abdominal pain, a randomized clinical trial

**DOI:** 10.1186/s40360-020-00403-8

**Published:** 2020-03-17

**Authors:** Masoud Mayel, Saleh Hamzeh, Salile Shahabi Rabori, Sareh Ghasemirad, Nasim Zamani, Hossein Hassanian-Moghaddam

**Affiliations:** 1 Department of Emergency Medicine, Kerman University of Medical Sciences, Kerman, Iran; 2 Social Determinants of Health Research Center, Shahid Beheshti University of Medical Sciences, South Karegar Street, Tehran, Iran; 3 Department of Clinical Toxicology, Loghman Hakim Hospital, School of Medicine, Shahid Beheshti University of Medical Sciences, Tehran, Iran

**Keywords:** Lead, Poisoning, Abdominal pain, Treatment, Calcium

## Abstract

**Background:**

In 2016, in a lead poisoning outbreak in Iran, physicians reported thousands of opium users who presented to emergency departments (EDs) with intractable severe abdominal pain which did not respond to any narcotic medication. During the same period of time, we investigated the efficacy of intravenous calcium gluconate in alleviating lead-induced abdominal pain.

**Methods:**

In a single-center, single blinded, randomized controlled trial, a convenient sample of adult opium-addicted patients who presented to an academic ED with abdominal pain and had an initial diagnosis of lead poisoning were included and randomly subjected to two treatment groups receiving conventional treatment (morphine 0.1 mg/kg + normal saline; group 1) and conventional treatment plus 1 g of intravenous calcium gluconate (group 2) to alleviate their abdominal pain. The visual analogue scale (VAS) was determined by each patient (0 to 100 mm) before treatment, and 15, 30, and 60 min after intervention.

**Results:**

A total of 50 patients (25 in each group) were enrolled. Blood lead levels, VAS scores before treatment, and mean administered dose of morphine were similar between the two groups. After treatment, mean VAS score dropped to 64.7± 10.4 vs. 67.1± 10.9 at 15 min (*P* = 0.437), 64.6± 10.9 vs. 58.0 ± 11.2 at 30 min (*P* = 0.041), and 63.8± 10.7 vs. 53.6± 10.9 at 60 min (*P* = 0.002) in groups 1 and 2, respectively.

**Conclusion:**

Intravenous calcium gluconate administration along with morphine can improve abdominal pain in lead poisoning due to the ingestion of lead-contaminated opium. Further interventional studies are recommended to see if response to calcium salts in suspected lead-induced abdominal pain can rule in lead toxicity.

**Trial registration:**

IRCT20171009036661N2. Registered 27 May 2018 - Retrospectively registered,

## Background

Abdominal pain is one of the major chief complaints in patients who refer to toxicologists due to lead poisoning [1]. The crampy and diffuse abdominal pain may accompany with constipation or diarrhea; however, the pathogenesis of lead colic remains unclear. This presentation may mimic a variety of surgical and nonsurgical diseases [2]. Changes in the visceral smooth muscle tone through lead action on the visceral autonomic nervous system, alterations in sodium transport in the small intestinal mucosa, lead-induced interstitial pancreatitis, and spasmodic contractions of intestinal wall smooth muscles have been suggested as the most possible causes of abdominal pain in lead-poisoned patients [3].

It is generally said that severe colicky pain is rare in lead-poisoned patients [2]. However, during the recent lead poisoning outbreak in Iran in 2016 (due to ingestion of lead-contaminated opium), physicians anecdotally reported thousands of opium users referring to emergency departments (EDs) throughout the country with severe abdominal pain that did not respond to routine treatments of abdominal pain including narcotics [4]. Abdominal pain was so severe in some cases which necessitated surgical exploration for possible acute abdomen [5]. In fact, abdominal pain was severe enough and resistant to routine treatments in these patients which made us think about other possible adjuvant treatments.

It is generally advocated that some of the lead poisoning signs and symptoms are due to its chemical similarity to divalent cations calcium, magnesium, and zinc. Lead interferes with numerous calcium-mediated metabolic pathways particularly in mitochondria and in second-messenger systems regulating cellular energy metabolism [6]. Lead also functions as an antagonist or agonist of calcium-dependent processes [7]. Although these changes have been mentioned in neurologic pathways, they can theoretically be involved in any calcium-mediated metabolic pathway throughout the body.

With such concept, we tried to evaluate the efficacy of intravenous (IV) calcium gluconate in treatment of lead-induced abdominal pain which was the main goal of the current study.

## Methods

In a randomized, single-center, single-blinded, parallel group trial (1:1 allocation ratio), we compared the efficacy of morphine versus morphine plus IV calcium gluconate in reversal of abdominal pain due to lead poisoning in the patients who had referred with lead-contaminated opium induced lead poisoning. The study was performed in a single academic center in Kerman, Iran, between September 2016 and March 2018. The trial was submitted to and approved by Iranian Clinical Trial Registry (IRCT20171009036661N2). All patients provided informed consents before inclusion.

### Trial population

During a lead poisoning outbreak among opium users in Iran [4], all adult (older than 18 years of age) orally opium-dependent patients with blood lead levels (BLLs) higher than 25 μg/dL admitted to a single academic ER in Kerman who had acute abdominal pain were included into the trial. The referral center was in south of Iran with the highest percentage of opium abusers and dependents (17.1 and 5.3%, respectively) in the region [8]. Patients were randomly assigned into two groups (25 patients in each group) using random allocation sequence with SAS statistical software. We used sequentially numbered, sealed opaque envelopes for allocation concealment.

BLLs had been measured before ED presentation in medical clinics in an attempt to find the cause of anemia, weakness, constipation, neurological symptoms, or previous chronic abdominal discomfort using atomic absorption technique. Thus, the initial impression was lead toxicity in ED, unless an acute abdomen was detected by emergency physician and general surgeons.

**Fig. 1:**
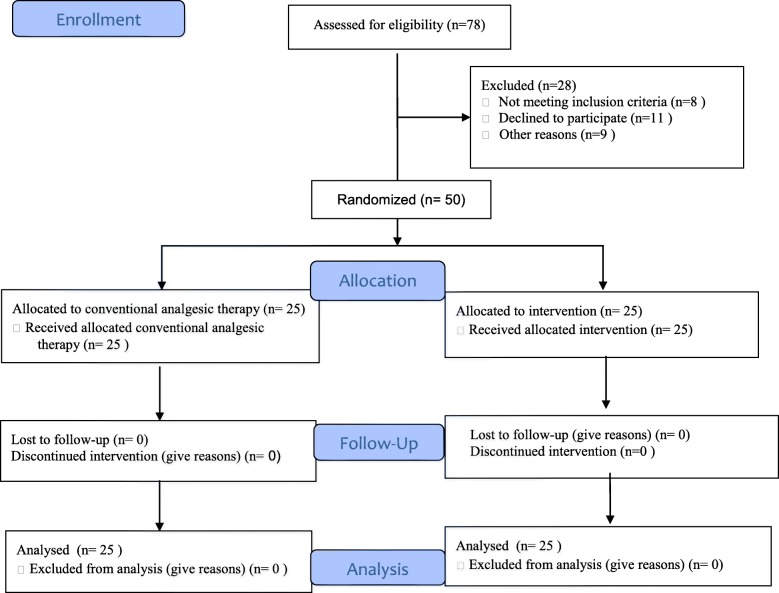
CONSORT Flow Diagram

Patients had to be on regular opium or its natural derivatives consumption for at least 3 months. They were excluded from the trial if they had been suffering from mental retardation, neuropathic diseases (i.e. diabetes), chronic pain syndromes, hypertension and cardiac disorders, hypochondriasis, contraindications for calcium gluconate injection (i.e. hypersensitivity to this group of drugs), kidney stones, hypercalcemia, hypophosphatemia, pregnancy, asthma, obstructive bowel obstruction, acute abdominal symptoms and acute abdomen, any confirmed diagnosis other than lead poisoning, using psychoactive drugs, and chronic severe history of morphine sensitivity (CONSORT Diagram; Fig. 1). Patients were characterized at baseline by age, sex, visual analogue scale (VAS) score, and BLL (Table 1).

**Table 1:** On-arrival characteristics of lead-poisoned patients with abdominal pain (*N* = 50)

Variable	Group 1 (*n* = 25)	Group 2 (*n* = 25)	P
Mean ± SD age (y) (min, max)	48.5 ± 11 (29, 73)	47.9 ± 10.3 (28, 70)	0.843^a^
Median [IQR] Daily opium (gr) (min, max)	8 [7.7, 10.5] (7, 12.5)	9 [7.7, 10.5] (7, 12.5)	0.569^b^
Mean ± SD Blood Lead Level (μg/dL) (min, max)	59.2 ± 22.2 (25, 92)	56.8 ± 16.6 (29, 91)	0.673^a^
Median [IQR] Morphine (gr) administered (min, max)	7 [6.5, 9] (6, 10)	7.5 [6.7, 9] (6, 10)	0.569^b^
Median [IQR] Amylase (min, max)	80 [46, 88] (6, 130)	70 [42, 88] (22, 102)	0.443^b^
Mean ± SD MCH (min, max)	24.5 ± 1.6 (22, 27)	25.2 ± 3 (18.9, 30)	0.324^a^
Mean ± SD MCV (min, max)	81 ± 5.4 (70, 97)	82.5 ± 8 (69.5, 103.5)	0.449^a^
Median [IQR] Hgb (min, max)	10 [9, 10.6] (8, 15.9)	10 [8.7, 11.4] (7.2, 16.8)	0.655^b^
Mean ± SD WBC (min, max)	6868 ± 1344 (4400, 9400)	7172 ± 1603 (4200, 9800)	0.471^a^
Mean ± SD VAS Score (min, max)	67 ± 10.3 (50, 83)	69.3 ± 11.6 (50, 90)	0.464^a^

^a^Student T-test, ^b^Mann Whitney U test

### Sample size

To provide 90% power at a 0.05 significance level (2-sided test) and to detect a VAS score difference in the primary endpoint of 10 mm (from mean of 65 to 55) on the 100-mm VAS with a standard deviation (SD) of 10 mm in a paired sample analysis, 22 patients were measured for each group. Considering 20% dropout, 25 patients were recruited in each arm.

### Trial design

A flow diagram of the trial is shown in Fig. 1. Our study adheres to CONSORT guidelines. The trial consisted of three time frames; an enrollment period, a blind treatment period, and 15-, 30-, and 60-min follow-ups. During the enrollment period, patients’ eligibility including high BLL, abdominal pain, and the VAS score was assessed.

Patients were randomized on admission to ED by first author using a block randomization list to two treatment arms: a slow IV morphine arm (0.1 mg/kg as the basic analgesic dose; group 1) and the same morphine dose plus 10 mL intravenous calcium gluconate 10% (intervention group; group 2). These doses were chosen based on data from previous studies [1, 4]. VAS score was checked and recorded on admission and 15, 30, and 60 min after treatments were given in the two groups by co-authors. Changes in the VAS scores were then compared between the groups.

There was no changes to methods after trial commencement.

### Measurement, efficacy and safety assessment

The VAS score was determined by each patient (0 to 100 mm) before treatment, and 15, 30 and 60 min after the administration of the trial medications. Safety assessment was done using cardiac monitoring, pulse oximetry, and evaluating vital signs during ED stay. There was no change to trial outcomes after the trial commenced.

### Statistical analysis

The data was analyzed using Statistical Package for Social Sciences (SPSS; IBM Corp., Armonk, N.Y., USA) software version 25 by application of Kolmogorov Smirnov, paired-samples t-test in different time intervals, One-way ANOVA, Mann Whitney U test, and chi square test. Considering significant reduction in VAS pairwise comparison in each time interval (Zero versus 15, 15 versus 30, and 30 versus 60 min post intervention) as the censored in event status, a log rank test (Kaplan-Meier) was run to determine if there were differences in the VAS score distribution by adding calcium to opioid prescriptions. A *P* value less than 0.05 was considered to be statistically significant.

## Results

A total of 25 patients were evaluated in each group. All were male and mean age was 48.2 ± 10.5 years. On arrival characteristics are shown in Table 1 with no significant difference between the two groups. Severity of pain was also similar between the groups on presentation.

**Fig. 2:**
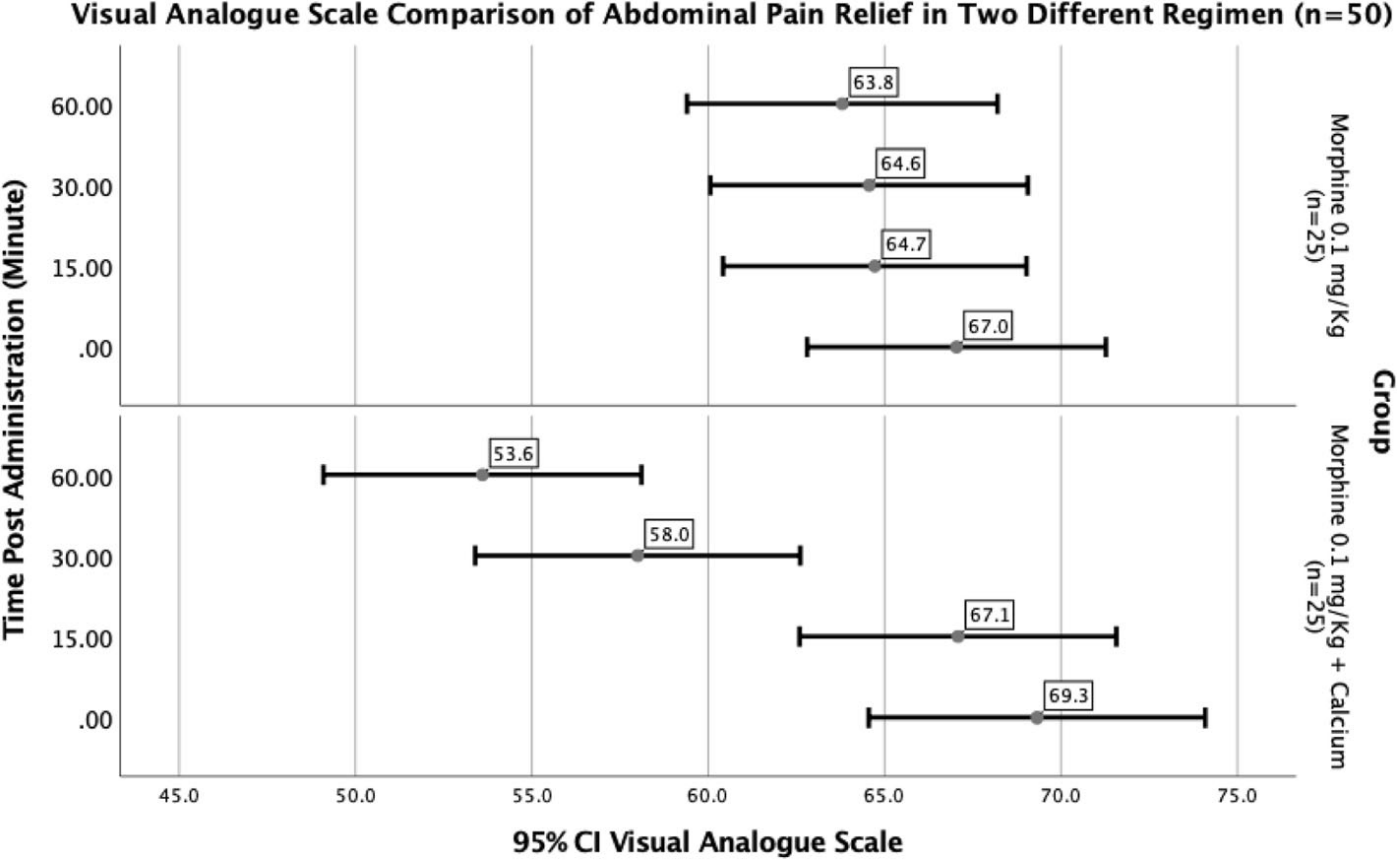
Visual Analogue Scale comparison of Abdominal Pain Relief in Two Different Regimen (*n* = 50)

The VAS score decreased to 67.1± 10.9, 58.0 ± 11.2, and 53.6± 10.9 at 15, 30 and 60 min after administration of morphine plus IV calcium gluconate in group 2, while these measures were 64.7± 10.4, 64.6 ± 10.9, and 63.8± 10.7 in the abovementioned time frames in group 1 which received solely morphine (all Ps < 0.001, Fig. 2). Analysis of the variance of VAS scales between the two groups at 15-, 30-, and 60- min intervals after treatment is also shown in Table 2.

**Table 2:** Analysis of Variance in different time intervals comparing the effects of morphine vs. calcium + morphine

		Sum of Squares	df	Mean Square	F	Sig
0	Between Groups	64.980	1	64.98	.544	0.464
	Within Groups	5730.400	48	119.38		
	Total	5795.380	49			
15 Minutes	Between Groups	69.620	1	69.62	.614	0.437
	Within Groups	5442.880	48	113.39		
	Total	5512.500	49			
30 Minutes	Between Groups	537.920	1	537.92	4.420	0.041
	Within Groups	5842.160	48	121.71		
	Total	6380.080	49			
60 Minutes	Between Groups	1300.500	1	1300.50	11.167	0.002
	Within Groups	5590.000	48	116.46		
	Total	6890.500	49			

Pairwise comparison of each group in three subgroups is mentioned in Table 3. Comparing the VAS distributions after adding calcium gluconate to conventional opioid analgesic program showed to have dramatic effect on alleviating the patients’ abdominal pain (χ^2^[2)] = 106.802, *P* < .0001).

**Table 3:** Pairwise comparison of 2 different arms of abdominal pain treatment in patients with lead toxicity (*n* = 50)

Group			Mean	N	Std. Deviation	Mean Paired Difference	95% CI of difference	Sig
**A (Morphine 0.1 mg/Kg)**	Pair 1	Zero minute	67.04	25	10.261	2.32	1.86, 2.78	>.001
		Fifteen minute	64.72	25	10.414			
	Pair 2	Zero minute	67.04	25	10.261	2.48	1.71, 3.25	>.001
		Thirty minute	64.56	25	10.901			
	Pair 3	Zero minute	67.04	25	10.261	3.24	2.11, 4.37	>.001
		Sixty minute	63.80	25	10.661			
	Pair 4	Fifteen minute	64.72	25	10.414	0.16	−0.53, 0.85	.637
		Thirty minute	64.56	25	10.901			
	Pair 5	Fifteen minute	64.72	25	10.414	0.92	−0.23, 2.07	.111
		Sixty minute	63.80	25	10.661			
	Pair 6	Thirty minute	64.56	25	10.901	0.76	−0.49, 2.01	.222
		Sixty minute	63.80	25	10.661			
**B (Morphine 0.1 mg/Kg + Calcium Gluconate)**	Pair 1	Zero minute	69.32	25	11.553	2.24	1.06, 3.42	>.001
		Fifteen minute	67.08	25	10.878			
	Pair 2	Zero minute	69.32	25	11.553	11.32	9.95, 12.69	>.001
		Thirty minute	58.00	25	11.162			
	Pair 3	Zero minute	69.32	25	11.553	15.72	14.26, 17.18	>.001
		Sixty minute	53.60	25	10.920			
	Pair 4	Fifteen minute	67.08	25	10.878	9.08	8.51, 9.65	>.001
		Thirty minute	58.00	25	11.162			
	Pair 5	Fifteen minute	67.08	25	10.878	13.48	12.85, 14.11	>.001
		Sixty minute	53.60	25	10.920			
	Pair 6	Thirty minute	58.00	25	11.162	4.40	3.85, 4.95	>.001
		Sixty minute	53.60	25	10.920			

## Discussion

Lead-induced abdominal pain was first introduced by Hippocrates in 370 B.C. Through the Middle Ages, outbreaks of lead-induced abdominal pain due to contaminated cooking utensils occurred in Europe. Lead-induced abdominal pain was noted to be common among lead workers and particularly severe after inhalation of lead fumes. Lead colic is often the symptom that brings the lead-poisoned patient to a physician [9].

The amount of lead exposure or blood lead level that causes abdominal pain is unknown but it seems that abdominal pain due to lead starts to occurs at blood lead levels ranging from 40 and 80 mg/100 mL [10]. Our study showed that even less BLLs may cause abdominal pain.

Administration of calcium to alleviate abdominal pain is somehow an old method of treatment that is not currently advocated in textbooks and recent articles. Shelling mentioned that lead colic might be alleviated, almost instantly after the intravenous administration of calcium chloride which was thought to add additional evidence that calcium “drives” lead into the bones [11]. However, the later studies of Aub and coworkers discredited such an assumption since the alleviation of pain is too rapid to be due to precipitation of lead in the bone tissue. Aub and his coworkers were, therefore, inclined to believe that the action of calcium in this instance is to relax the intestinal tract [12]. We also believe that this effect may be due to the effects of calcium on the intestinal cells which make these cells more relaxed. Our study also confirms the rapid onset of action of calcium in lead toxicity as many of our patients report improvement in their abdominal pain in 1 h following initiation of the treatment.

Another recent animal study mentioned that succimer used alone could reduce lead levels in blood and bone and reverse activities of aminolevulinic acid dehydratase in blood; however, a better therapeutic efficiency in mobilizing bone lead could be achieved by succimer used with calcium and ascorbic acid [13]. Our results are in accordance with these studies although again, the exact mechanism of alleviation of abdominal pain by calcium is not clear. Our study showed that although opioids were useful in alleviating pain, their sole administration might not be efficient enough in reversal of abdominal pain in these patients. The pain is better and faster managed by combination of IV morphine and calcium gluconate within the 15, 30 and 60 min post intervention. This is while with opioid administration alone, severity of pain does not change significantly after 15 min. In general, it seems that adding calcium to the analgesic regimen results in continuing relief of pain in different time intervals.

### Limitations

Our limited sample size is definitely a major shortcoming of the current study. On the other hand, we were unable to follow the patients in the ward to see if they needed more pain killers in the subsequent hours after admission. The patients were not provided with their maintenance equivalent dose of opioid to avoid withdrawal syndrome and this could increase their pain; however, this was similar between the two groups of the study.

## Conclusions

Intravenous calcium gluconate administration along with morphine can improve abdominal pain in lead poisoning due to the ingestion of lead-contaminated opium. Further studies on larger sample sizes are warranted to better elucidate this effect. Also, interventional studies are recommended to see if response to calcium salts in suspected lead-induced abdominal pain can rule in lead toxicity.

## Data Availability

All data generated or analysed during this study are included in this published article.

## Abbreviations

ANNOVA: Analysis of variance
BLL: Blood lead level
CONSORT: Consolidated standards of reporting trials
ED: Emergency departments
ER: Emergency room
IV: Intravenous
SAS: Statistical Analysis Software
SPSS: Statistical Package for Social Sciences
VAS: Visual analogue scale
